# Robotic devices for paediatric rehabilitation: a review of design features

**DOI:** 10.1186/s12938-021-00920-5

**Published:** 2021-09-06

**Authors:** Alberto Gonzalez, Lorenzo Garcia, Jeff Kilby, Peter McNair

**Affiliations:** 1 BioDesign Lab, School of Engineering, Computer and Mathematical Sciences, Auckland University of Technology, Auckland, New Zealand; 2 Health and Rehabilitation Research Institute, Auckland University of Technology, Auckland, New Zealand

**Keywords:** Robotic, Exoskeletons, Rehabilitation, Assistance, Children, Physical disability

## Abstract

Children with physical disabilities often have limited performance in daily activities, hindering their physical development, social development and mental health. Therefore, rehabilitation is essential to mitigate the adverse effects of the different causes of physical disabilities and improve independence and quality of life. In the last decade, robotic rehabilitation has shown the potential to augment traditional physical rehabilitation. However, to date, most robotic rehabilitation devices are designed for adult patients who differ in their needs compared to paediatric patients, limiting the devices’ potential because the paediatric patients’ needs are not adequately considered. With this in mind, the current work reviews the existing literature on robotic rehabilitation for children with physical disabilities, intending to summarise how the rehabilitation robots could fulfil children’s needs and inspire researchers to develop new devices. A literature search was conducted utilising the Web of Science, PubMed and Scopus databases. Based on the inclusion–exclusion criteria, 206 publications were included, and 58 robotic devices used by children with a physical disability were identified. Different design factors and the treated conditions using robotic technology were compared. Through the analyses, it was identified that weight, safety, operability and motivation were crucial factors to the successful design of devices for children. The majority of the current devices were used for lower limb rehabilitation. Neurological disorders, in particular cerebral palsy, were the most common conditions for which devices were designed. By far, the most common actuator was the electric motor. Usually, the devices present more than one training strategy being the assistive strategy the most used. The admittance/impedance method is the most popular to interface the robot with the children. Currently, there is a trend on developing exoskeletons, as they can assist children with daily life activities outside of the rehabilitation setting, propitiating a wider adoption of the technology. With this shift in focus, it appears likely that new technologies to actuate the system (e.g. serial elastic actuators) and to detect the intention (e.g. physiological signals) of children as they go about their daily activities will be required.

## Introduction

Mobility and exploration are essential in children’s development and contribute towards cognitive, physical, social and emotional development. However, children with physical disabilities present limitations when performing activities autonomously, which hinders their typical development [1]. Ongoing paediatric physical disability arose from many different causes, including neurological disorders like cerebral palsy (CP) [2], Stroke [3] and acquired brain injury (ABI) [4], neuromuscular diseases such as Duchenne muscular dystrophy (DMD) [5] and spinal muscular atrophy (SMA) [6], or traumatic injuries [7, 8] (Table 1).

**Table 1:** Incidence or prevalence of conditions that cause physical disabilities in children

Condition	Incidence or prevalence
Cerebral palsy	Prevalence of 1 per 500 live births [2]
Stroke	Incidence of 1.2 to 13 per 100,000 children per year [3]
Traumatic brain injury	Incidence of 691 per 100,000 children [7]
Duchenne muscular dystrophy	Prevalence of 1 per 5000 live male births [14]
Spinal muscular atrophy	Prevalence of 7.8–10 per 100,000 live births [7]

Rehabilitation is essential to help the children recover or maintain functionality when interacting with their environment, improving the quality of life and autonomy [9, 10]. Furthermore, early access to rehabilitation is critical for children while they are in the stage of development. The gait pattern and motor abilities are still malleable [11], intending to reduce the probability of developing more severe levels of disability [12, 13].

The standard therapies to manage the musculoskeletal system’s deterioration and improve and maintain physical ability include passive orthoses, surgery, and physiotherapy [15, 16]. Physiotherapists prescribe, monitor, and guide exercise, which can prevent an unnecessarily sedentary or immobile lifestyle. The most extensively investigated aspect of physiotherapy is the effect of direct interventions on upper or lower limbs. Such interventions often involve intensive stretching and strengthening exercises facilitated by the physiotherapist [2] to improve motor skills. These interventions are often highly labour intensive and can be challenging to perform [17]. Furthermore, the effectiveness of physiotherapy often depends on the experience of the physiotherapist. Thus, it is not easy to achieve optimal consistency and repeatability between rehabilitation sessions [18, 19].

There is a growing interest in robots that can support the patient, the family and the medical professional in a wide range of activities used for the care of people with physical disabilities, for example, companion robots [20, 21], monitoring robots [22] and surgery robots [23], all of them can be considered as healthcare robots.

Healthcare robots can be divided into three main categories, clinical robots, assistive robots and rehabilitation robots [24, 25]. Clinical robots are focus on supportive care and cure process (e.g. help in surgery and diagnosis) in clinical environments; assistive robots primary function is to provide assistive help either to carers or directly to patients either in a hospital or in a specialist care facility (e.g. patient lifting and to assist in routine services); rehabilitation robots are robots design towards restoring the functionality and mobility of people with physical disabilities, in that case, the recovery of mobility could be achieved by assisting the patient during ADLs (e.g. walking and grasping objects) [17, 26] or with physical training therapy [24, 27–30], and are the main focus of this study.

Rehabilitation therapy for the recovery of mobility based on robots has been proposed as a new procedure for children with physical disabilities [31]. This robot-assisted rehabilitation therapy consists of a mechatronic device that provides highly repetitive and task-specific guided movements autonomously [32, 33]. The use of robots in rehabilitation therapies bring advantages over traditional therapies, as they allow extensive practice in children with substantial disabilities, reduced effort required of therapists during the exercises, and provide a quantitative assessment of the patient’s motor function (e.g. quantitative feedback of range of motion (ROM) and strength with each repetition) [34–38].

Rehabilitation robots are often classified by their mechanical structure and are generally divided into end-effectors and exoskeletons [28, 39]. End-effector devices work by applying forces to the distal segments of limbs, creating what is termed a “mechanical chain” that prompts movements of other parts of the limb generating a pattern of specific activity across different joints. If utilised on a single segment and joint, their simple structure makes it easier to adapt them to many patients and needs less complicated control algorithms. However, it is difficult to isolate specific joints since they produce complex movements that involve the whole limb [40].

Contrarily, robotic exoskeletons could be termed “wearable machines” that mirror the patient’s skeletal structure; therefore, they only move the joint of the limb where the exoskeleton is worn. This approach allows for independent and concurrent control of specific segments of the limb. However, it is essential to adjust the length of sections of the robot to the lengths of the segments of the patient limb. Moreover, when the joint is in motion, the position of the centre of rotation can change, creating discomfort in the user. Thus, increasing the number of degrees of freedom of the robot increases the control algorithm’s complexity, weight, mechanical complexity, and power requirements, making it unattainable for home use [18, 41].

Apart from mechanical structure, robots possess essential elements to ensure the systems’ reliability and robustness [42]. Actuators, training strategy and the Human–computer interface (HCI) are among these essential elements. The actuators play a crucial role because they determine the torque and movement provided by the robot and influence the total weight and compliance of the system [29, 43]. The training strategy and the HCI are an integral part of the robot-assisted rehabilitation since it determines how the patient interacts with the robot and the type of assistance that the robot can provide. Many authors have analysed these last two characteristics as part of the robots control [28, 30, 44]. However, control also involves “low level” considerations that are more related to the internal communication of the components (sensors, structures, microcontrollers, actuators, etc.) at a hardware level rather than how the device interacts with the patient [45–49].

Although multiple devices for the robotic rehabilitation of upper and lower limbs have been developed, at least in a proof-of-concept phase [24, 39, 42, 44, 45, 50, 51], most presented robots were designed for adult users, impeding their use on the paediatric population. For example, commercial exoskeletons are made for a subject 150 cm tall onwards [52], while the average height for a 5-year-old child will be around 110 cm [53].

However, to develop technology planned to be used on the paediatric population is not only a matter of reducing the size of the robots. But it should be tailored to their own capabilities and goals that differ from those of the adults. For instance, a simple downscaling of the robots is not enough as the normalised joint torques on adults are greater than those of a child [54, 55], making them potentially dangerous when used on small children. Additionally, in the case of children, as their cognitive abilities are still developing, it could be hard for them to fully understand how the technology works [52, 56]. Hence, it is hard to adapt a robot made for adults to be used by children since the robots do not fulfil the children’s needs [26, 57].

Consequently, to address the children’s needs adequately, it is essential to include them and other stakeholders (e.g. family members, clinicians, and health care providers) during the development process, providing feedback to identify possible issues of importance [1, 43]. Furthermore, it is essential to focus not only on addressing the impairment or limitation in users’ functional abilities, but also on other fundamental needs, like accessibility and aesthetics [58], to avoid the user abandoning the rehabilitation device due to frustration [59].

Despite the progressive development of robotic rehabilitation devices, their application to the paediatric population is still scarce. Consequently, the key features to design an optimal robotic rehabilitation device that better enhance children’s abilities with physical disabilities have not been well defined yet. Based on this framework, this review aims to address the following questions: (1) What are the design requirements for paediatric rehabilitation robots? (2) How does the current technology contribute to achieving the paediatric design requirements? And (3) How do the paediatric conditions impact the device design?

## Methods

An in-depth literature search was performed to conduct the review, following the search strategy of the Preferred Reporting Items for Systematic Reviews and Meta-Analyses (PRISMA) guidelines [60].

A literature search was conducted to identify literature associated with the topic based on searches in PubMed, Scopus, and Web of Science, using the combination of the following keywords: (pediatric OR kid* OR child*) AND (aid OR assist* OR improve* OR augment* OR enhance* OR reinforce* OR therap* OR rehabilitation) AND (active ortho* OR exoskeleton* OR wearable robot* OR portable robot* OR robot* suit OR robot*) AND (movement OR motion OR walk* OR gait OR grasp* OR handl*). To make our search as complete as possible, a search through the university library databases was also conducted.

After the preliminary search, the following inclusion and exclusion criteria to narrow the literature search were used. The inclusion criteria were:

1. Studies involving robotic devices for robot-assisted rehabilitation therapy,
2. Studies involving robotic devices for assessment of patients with Physically disabilities,
3. Studies involving devices designed for children or utilised with a paediatric population (< 18 years old),
4. written in English,
5. full-text articles.

And the exclusion criteria were:

1. Studies that only present software solutions or simulations,
2. Studies involving passive devices (do not have actuators),
3. Studies involving postural change,
4. Studies involving only the adult population and
5. Studies involving robots that do not replace the movement itself (e.g. wheelchairs).

### Findings

The outcome of this literature review is compiled in the following sections:

- An overview of the literature search,
- the paediatric robotic rehabilitation design requirements,
- an analysis of the type of robots used in paediatric robotic rehabilitation;
- the actuators to drive the robots;
- training strategy of the robots;
- the human–computer interface of the assistive systems, and
- the treated conditions in children with physical disabilities.

### Literature search

Based on the keywords mentioned in the methods section, 1604 publications were found, with:

- 811 publications from Web of Science,
- 547 publications from PubMed,
- 241 from Scopus, and
- 5 from a search on the University library.

**Fig. 1:**
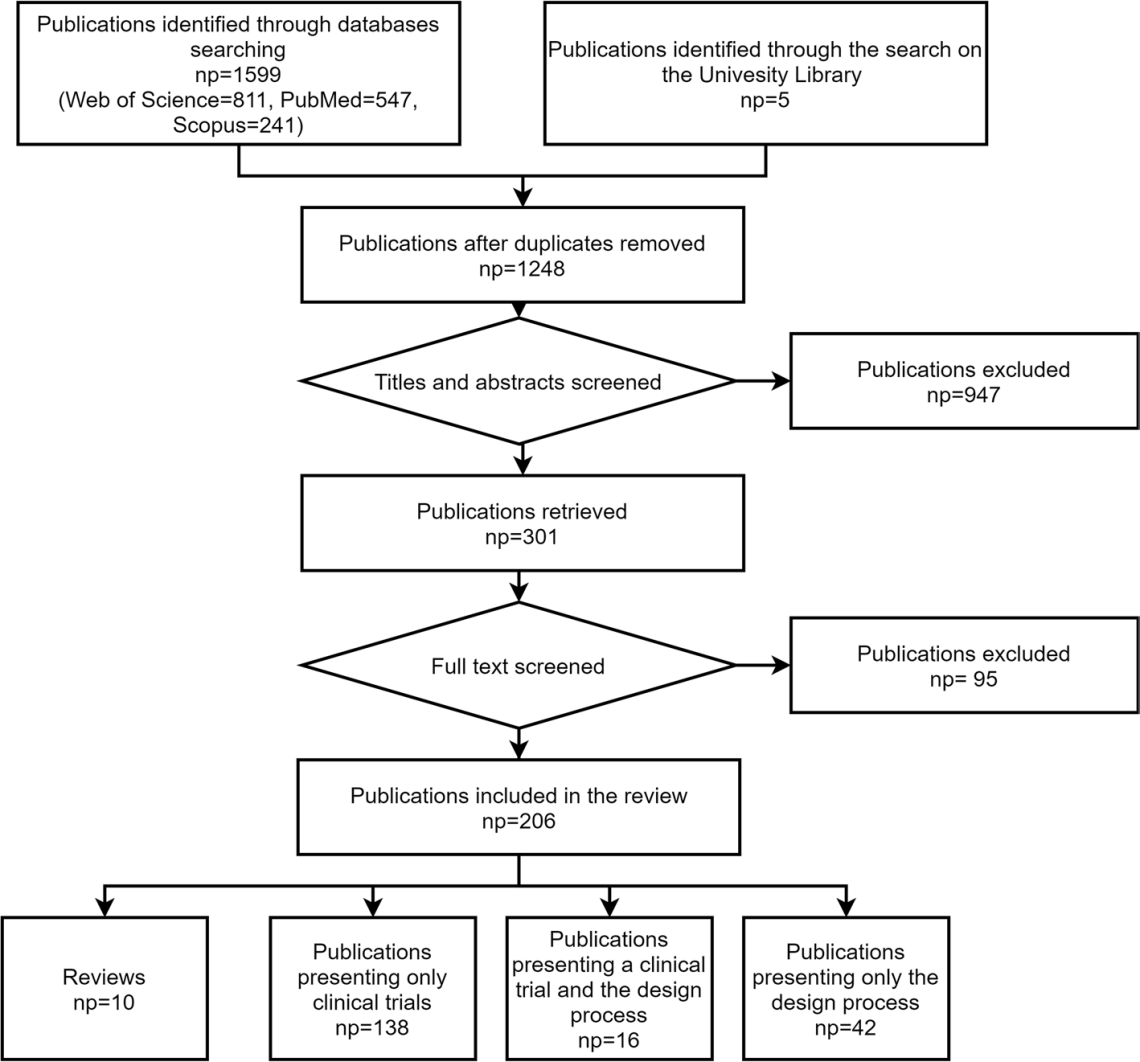
Literature search flow diagram

First, a check was made for duplicated publications. After this process, the abstracts of 1248 publications were screened, and 301 titles were selected for full-text reading. After carefully applying the inclusion–exclusion criteria to the full read papers, 206 publications were selected. Among the chosen publications, 10 were reviews, 42 only discussed a section of the design process of the rehabilitation robot, 138 presented a clinical application, and 16 included the design process plus a clinical application (Fig. 1 shows a flow diagram that illustrates the process of the selection of the papers).

The ten review articles examined a variety of rehabilitation robots for children with physical disabilities. They were focused on children with neurological problems (e.g. CP, ABI, and Stroke) or SCI and only investigated their use as part of physical therapy. In Fasoli et al. [35], Meyer et al. [61], and Bayon et al. [62], the robot assistive therapies for children with CP were examined. Vova et al. [63] reviewed the efficacy of functional electrical stimulation and exoskeletons in gait training to improve motor function and gait pattern in children with CP. Zwicker et al. [64] reviewed the efficiency of robot-assisted treadmill training compared to traditional treadmill training in children with CP. Chen et al. [65] examined the effectiveness of various devices for upper limb robotic therapy on children with CP. The effects of robotic gait training practices in individuals with CP were investigated in Carvalho et al. [31]. Falzarano et al. [10] and Mahamud et al. [66] investigated upper and lower limb rehabilitation devices for neurological diseases. Dannenberg et al. [67] compared different locomotor training, including robotic training, in children with SCI. Compared with the previous reviews, this work analyses a broader range of aspects of paediatric rehabilitation robots, focused on the design parameters to fulfil the paediatric needs and how the technology and different conditions affect the robot design.

### Paediatric robotic rehabilitation design requirements

Fifteen different requirements were identified (Table 2). The requirements are based on those proposed by Batavia and Hammer for assistive devices [68] and expanded by proposed requirements for paediatric rehabilitation devices highlighted by Weightman et al. [69], Bützer et al. [26] and Keller et al. [57]. In paediatric rehabilitation, it was apparent that the stakeholder’s needs related to operability, weight, safety, and motivation factors were relevant.

**Table 2:** Paediatric rehabilitation robots’ requirements and examples

Requirement	Definition	Example
Target group	Range of ages and problem of the users	ChARMin covered an age range from 5–18 years old [99]
Mechanical functionality	The device performance, including the controlling level of assistance, the functional workspace, smoothness of movement and robustness	McDaid designed a gait trainer that allows children to stretch their legs through the entire ROM and support body weight up to 80kg [40]
Weight	Total unsupported or unpowered mass of the device in relation to the user’s body weight	Lerner developed a Bowden cable structure for an ankle exoskeleton with a weight of 1.85 kg and placed 65% of the total mass above the waist to minimise the metabolic cost of walking due to the device's weight [73]
Therapeutic benefit	The type of exercise that the rehabilitation system should promote and how this will improve the user quality of life	The paediatric Anklebot provided intensive task-specific sensorimotor therapy to the ankle of children with motor disabilities to promote motor learning [75]
Safety	The potential for the device to harm its user	IOTA device included a security stop button that immediately halts the servo motors [175]
Comfort	The user can use the device without physical pain or discomfort	The P-LEG robot used 3D printed braces based on 3D scans of the child’s legs to improve the child's comfort [71]
Reliability	The consistency of the device operation in normal operating conditions	Laubscher designed a gait guidance controller to guide the motion of the patient's legs to follow healthy gait patterns to avoid unnatural gait patterns [176]
Operability	The device is easy to control and adaptable to changes in the user’s ability and sizes	ATLAS exoskeleton used a slide and tubular regulation size system to adapt to the fast growth of the patients at all stages [177]
Product appeal	User satisfaction with the design, like fit, appearance, and sound of the device	One of the main requirements for PEXO was an appealing design, so the kidPexo version resembles a crocodile [26]
Quality of construction	Typical use and care should cause no damage, distortion, or hinder the expected useful lifetime of the device	PEXO device did not have electronics in the hand module, making the device water and dustproof [26]
Social acceptability	Matches user needs for discretion or attention to avoid stigmatisation	Weightman selected the handgrip of his robot through a questionnaire with different aspects like shape, style, feel, and colour [69]
Motivation	Encompass any aspect of the device considered to motivate the child	ChARMin used an Audio-visual interface with various game-based virtual reality scenarios to motivate the child for active participation [57]
Cost	The financial burden of the initial purchase and ongoing costs of the device	Volpini developed a low-cost robotic gait trainer to be used in developing countries [87]
Easy to maintain/repair	The ease of keeping the device fully operational, including when damaged	P-Legs' brace 3D print fabrication method made it easy to get new braces as the children grow [71]
Portability	The possibility of the device to be transported between locations	Cleary developed a smaller version of Pedbot that can be used at home [153]

In paediatric rehabilitation devices, operability is critical as children are in a continuous development phase during which their bodies, cognitive capabilities and physical abilities (e.g. skill levels) are changing, making them a “heterogeneous population” [52, 70]. Consequently, the device must adapt to different children’s abilities and sizes [71].

An important consideration is that the robot’s weight could obstruct the movement pattern of the limb and increase the child’s energy consumption [72, 73]. Furthermore, due to their musculoskeletal system’s immature development, their muscle strength and joint torque generation may not be adequate to assist in the movement being undertaken [74].

Concerning safety, children often cannot adequately assess the hazards of using complex technological devices [56]. Therefore, it is crucial to design safety mechanisms that minimise risky situations. These should be able to be activated remotely by adults with the child [57]. Furthermore, the use of compliant materials with shock-absorbing features (e.g. elastic elements like spring and Bowden cables) would be beneficial [17].

Finally, motivation is crucial because function recovery is not enough to engage children in the rehabilitation process [75]. Consequently, researchers have used strategies to engage children, like aesthetic designs attractive to the children [26, 76] or a virtual environment where they can interact with virtual objects [77, 78].

### Type of robots used on paediatric robotic rehabilitation

**Fig. 2:**
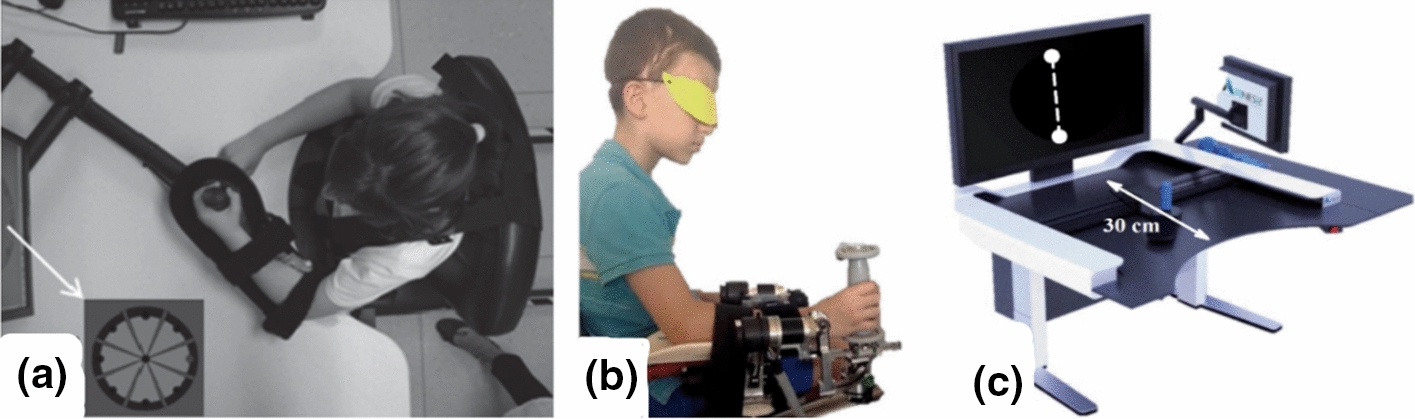
Picture of upper limb end-effectors rehabilitation robots: **a** Inmotion2/Mitmanus [79], **b **wrist robot [80], **c** REAPlan [81]

**Fig. 3:**
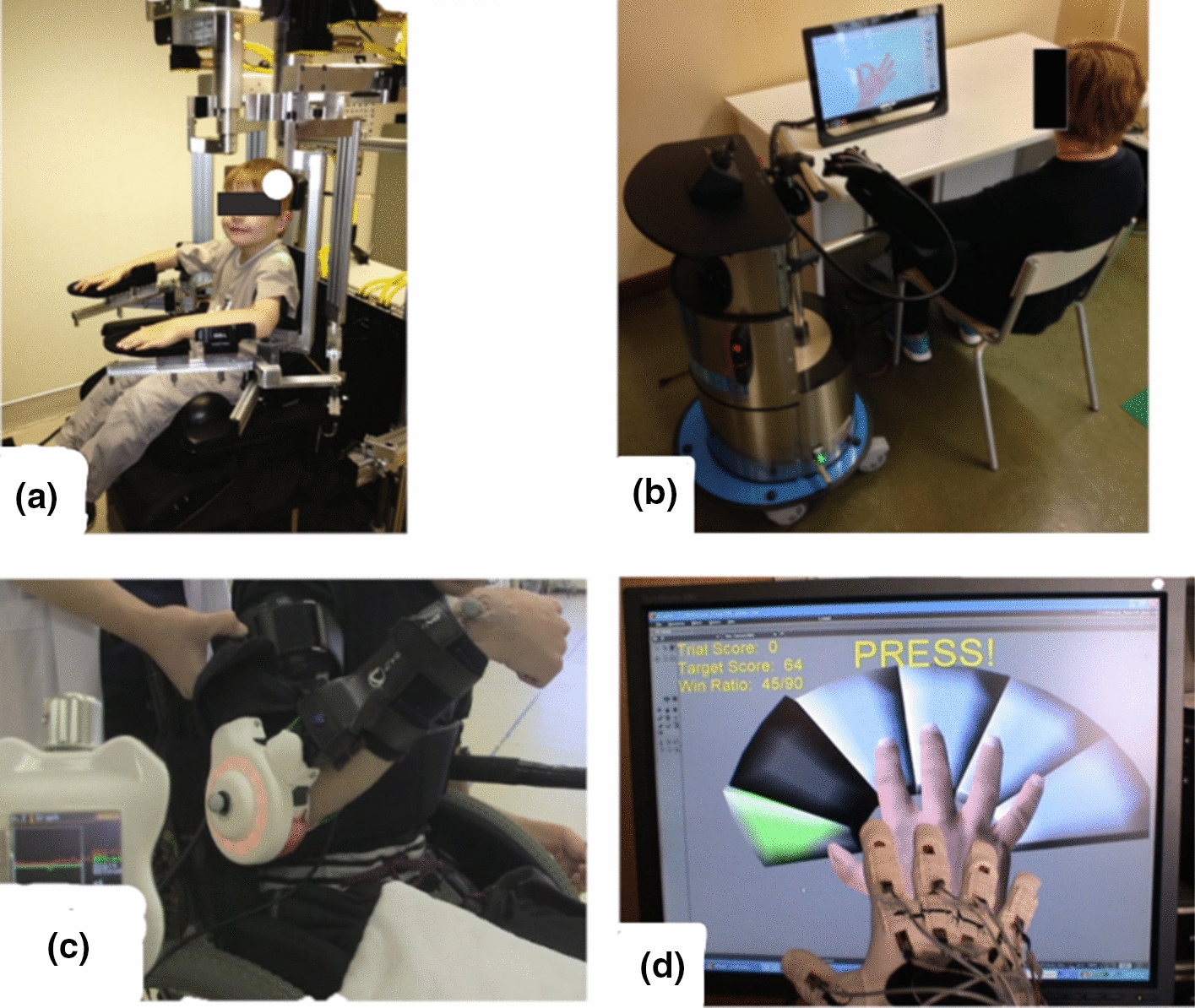
Picture of upper limb exoskeletons rehabilitation robots **a** KINARM [82], **b** GLOREHA [83], **c** HAL single joint [84], and **d** PneuGlove [85]

**Fig. 4:**
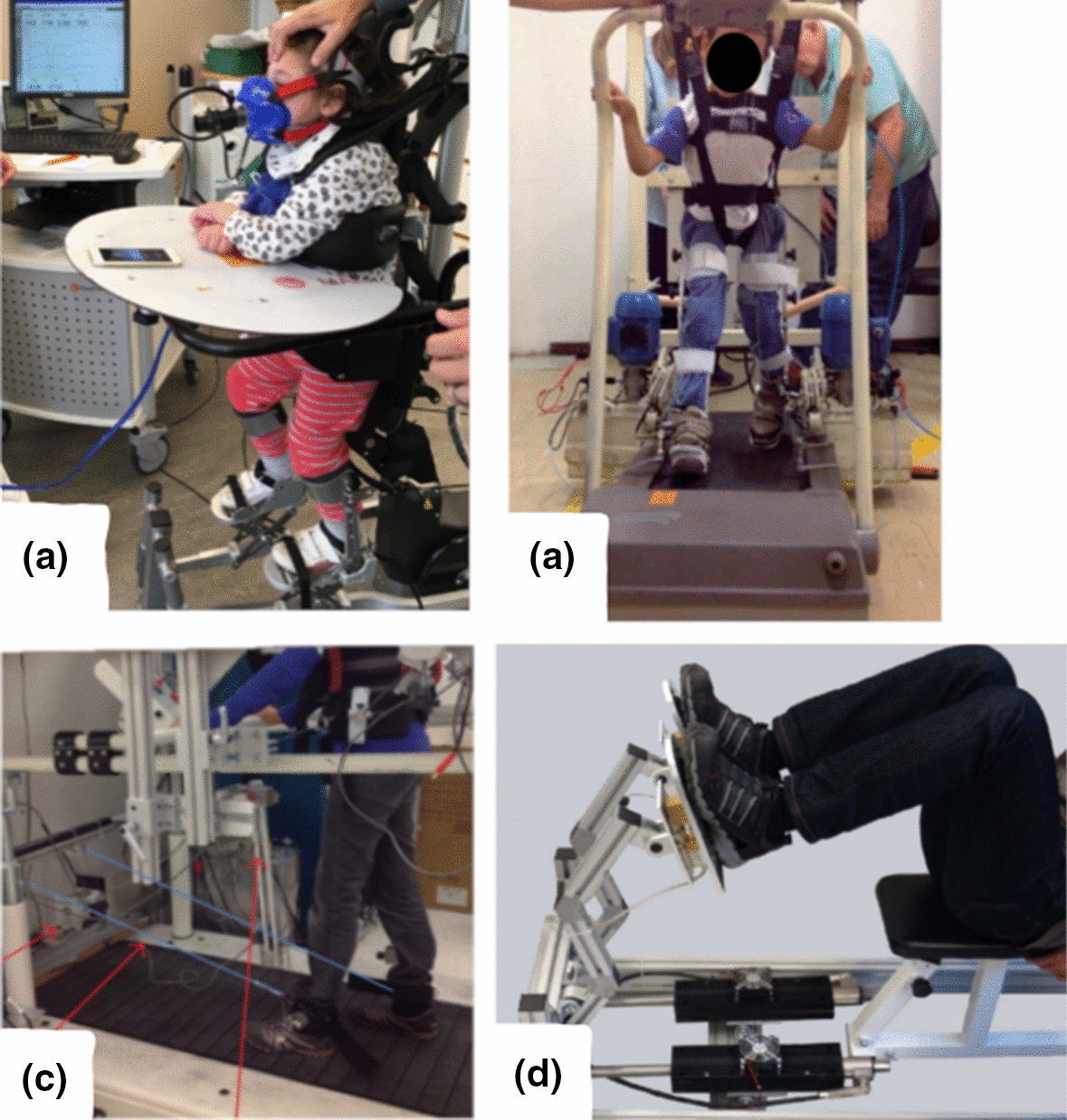
Picture of lower limb end-effectors rehabilitation robots: **a** Innowalk [86], **b** UFMG [87], **c** 3DCaLT [88], **d** Leg Press [89]. Reprinted from Biomedical Signal Processing and Control, Vol. 38, F. Chrif et al., Control design for a lower-limb paediatric therapy device using linear motor technology, Page 121, Copyright (2017), with permission from Elsevier

**Fig. 5:**
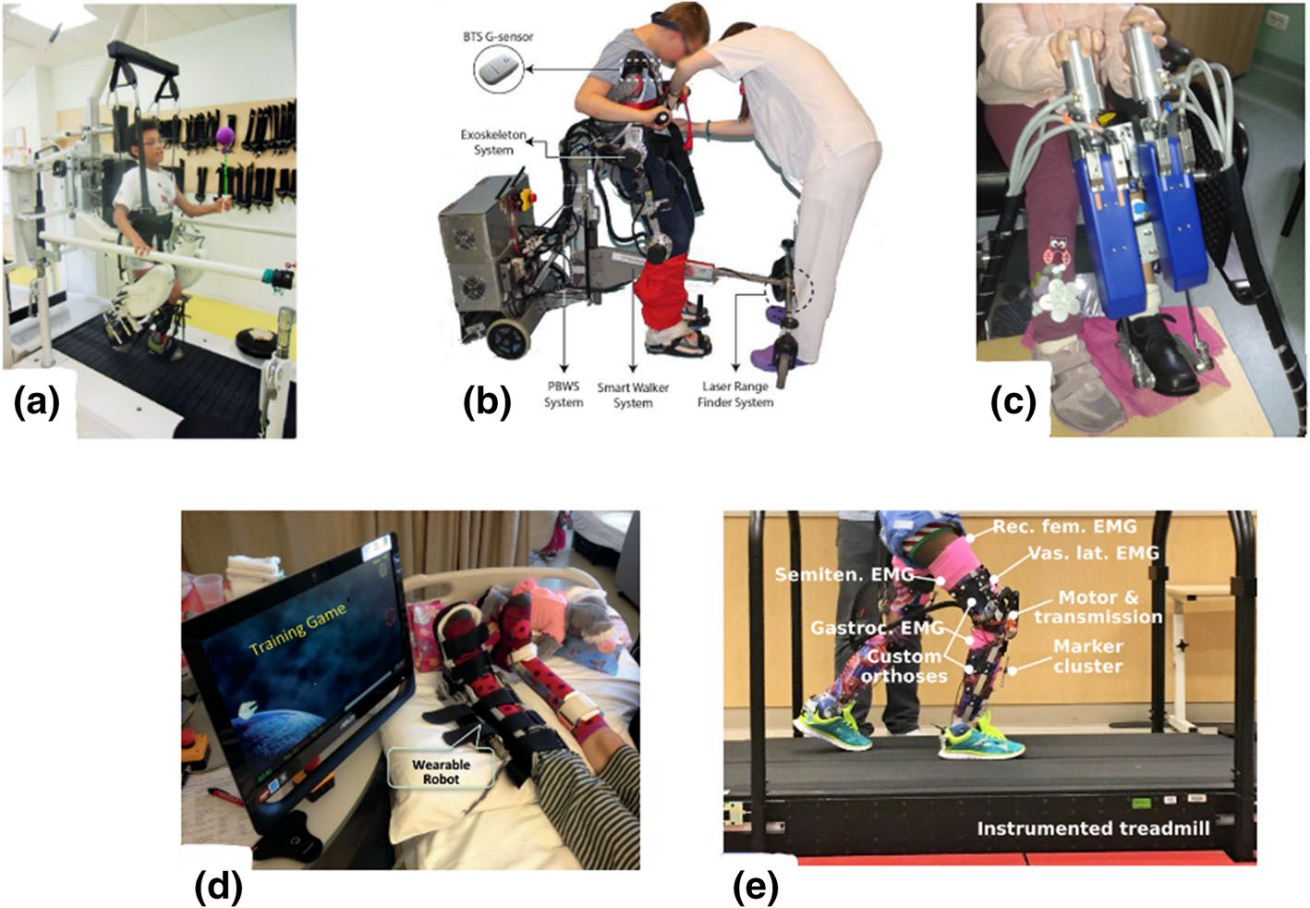
Picture of lower limb exoskeletons rehabilitation robots: **a** Lokomat [90], **b** HAL [91], **c** CPWalker [92], **d** PediAnklebot [93], **e** wearable ankle rehabilitation robot developed by the Rehabilitation Institute of Chicago [94], **f** P.REX [95]

**Fig. 6:**
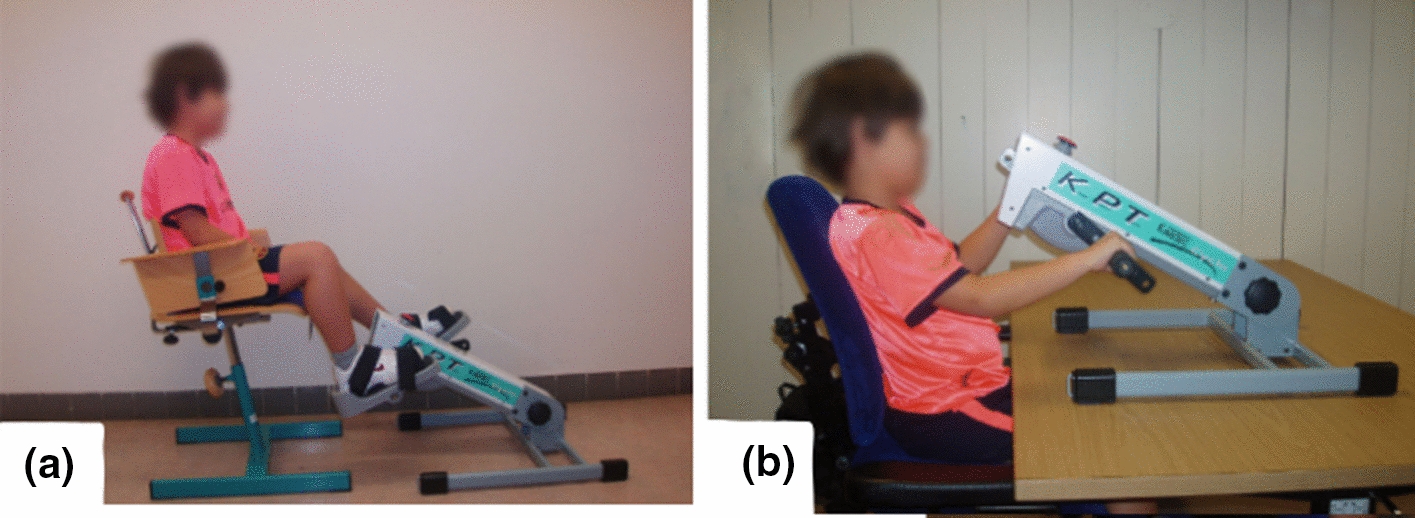
Picture of KPT Cycla [96] an end-effectors rehabilitation robot for both **a** lower and **b** upper limbs

Fifty-eight different devices were found that at least had a prototype in action. In Tables 3, 4, 5, 6, 7 (Figs. 2, 3, 4, 5, 6), the rehabilitation robots are presented chronologically and separated by their mechanical structure (end-effector or exoskeleton) and the anatomical part of the body where they are working (upper limb or lower limb). Furthermore, the tables summarise the characteristic features of the selected devices. This tabulated summary constitutes the reference for information provided in subsequent sections.

**Table 3:** Upper limb end-effectors rehabilitation robots

System (year)	Treated part of the body	DOFs	Actuator	Type of rehabilitation	Type of training	HCI input	Paediatric disease (design for or treated condition)	Paediatric design	Stage of the device
Inmotion2/Mitmanus (BIONIK, Canada) (1998) [126, 178]	Shoulder/elbow	2	DC motors	Physical therapy	Passive/active/assistive	Impedance	Neurological	No	Clinical trial/commercial (FDA)
Wrist-Robot (2009) [119, 179]	Forearm/wrist	3	DC motors	Physical therapy	Passive/active/assistive	Impedance	Neurological	No	Clinical trial
NJIT-RAVR (2009) [124, 180]	Shoulder/elbow/forearm	6	DC motors	Physical therapy	Active/assistive/resistive	Admittance	Neurological	No	Clinical trial
GNO arm (2009)[149]	Elbow	1	DC motor/Cable driven	Assistance	Assistive	Finger movement	DMD	Yes	Feasibility study
AMADEO (Tyromotion, Austria) (2012) [150, 181]	Fingers	5	DC motors	Physical therapy	Passive/active/assistive	Impedance	Physical disabled children	No	Clinical trial/commercial (FDA)
REAplan (AXINESIS, France) (2012) [81, 182]	Shoulder/elbow	2	DC motors	Physical therapy	Passive/active/assistive	Position	Neurological	No	Clinical trial/commercial
PASCAL (2013) [183, 184]	Shoulder/elbow	3	Dc motors	Physical therapy	Passive/active/assistive	Velocity	Neurological	Yes	Clinical trial
ReHaptic (2014) [125, 185]	Forearm/wrist	2	DC motors	Physical therapy	Passive/active/assistive/resistive	Admittance	Neurological	Yes	Clinical trial
MyPam (2015) [166]	Shoulder/elbow	2	Electric motors	Physical therapy	Active/assistive	Position	CP	Yes	Feasibility study

**Table 4:** Upper limb exoskeleton rehabilitation robots

System (year)	Treated part of the body	DOFs	Actuator	Type of rehabilitation	Type of training	HCI input	Paediatric disease (design for or treated condition)	Paediatric design	Stage of the device
KINARM (KinArm, Canada) (1999) [82, 186]	Shoulder/elbow	2	DC motors	Physical therapy	Passive/active	–	Neurological	No	Clinical trial/commercial
IOTA (2013) [175]	Thumb	2	DC motors/cable driven	Physical therapy	Passive/active/assistive	Movement	Neurological	Yes	Prototype
ChARMin (2014) [57]	Shoulder/elbow/wrist	6	Electric motors	Physical therapy	Passive/active/assistive	Movement	Neurological	Yes	Feasibility study
Universidad Nacional de San Juan (2014) [130]	Elbow	1	DC Motor	Physical therapy	Passive/assistive	EMG	Injuries	Yes	Clinical trial
Milwaukee University (2014) [187]	Wrist	2 actuated + 2 passives	DC motors/cable driven	Physical therapy	Assistive	Position	CP	Yes	Prototype
GLOREHA (2016) (IDROGENET, Italy) [33, 107]	Hand	5	Pneumatic	Physical therapy	Passive/active/assistive	Movement	Neurological	No	Clinical trial/commercial (FDA)
HAL single joint (Cyberdyne, Japan) (2019) [84]	Elbow	1	DC motor	Physical therapy/assistance	Assistive	EMG	CP	No	Clinical trial/commercial
PEXO (2019) [26]	Hand	2 Actuated + 1 passive	DC motors/cable driven	Physical therapy/assistance	Passive/assistive	Push buttons or EMG	Neurological	Yes	Feasibility study
PneuGlove (2019) [113]	Hand	5	Pneumatic	Physical therapy	Active/assistive/resistive	Movement	CP	Yes	Feasibility study
Exohand-2 (Android Technics, Russia) (2020) [131]	Hand	2 Each hand	Electric motors	Physical therapy	Assistive	EEG	CP	No	Clinical trial/commercial

**Table 5:** Lower limb end-effectors rehabilitation robots

System (year)	Treated part of the body	DOFs	Actuator	Type of rehabilitation	Type of training	HCI input	Paediatric disease (design for or treated condition)	Paediatric design	Stage of the device
GAIT trainer GT 1 (REHA-STIM MEDTEC, Switzerland) (2000) [188, 189]	Hip/knee	2	DC Motors	Physical therapy	Passive/assistive	Velocity	Neurological	No	Clinical trial/commercial
MOTOmed gracile (RECK, Germany) (2000) [190, 191]	Hip/knee	2	DC motors	Physical therapy	Passive /active/assistive/resistive	Velocity	Physical disabled	Yes	Clinical trial/commercial (FDA)
IntelliStretch (Rehabtek, USA) (2002) [192, 193]	Ankle	1	DC motor	Physical therapy	Passive/active/assistive/resistive	Velocity and torque	Neurological	No	Clinical trial/commercial (FDA)
Innowalk (Made for Movement, Norway)(2009) [114, 194]	Hip/knee	2	Electric motors	Physical therapy	Passive	–	Neuromuscular problems	Yes	Clinical trial/commercial
National Taiwan University (2009) [195]	Hip/knee	2	DC motors	Physical therapy	Passive	–	CP	Yes	Prototype
3DcaLT (2011) [134, 196]	Hip/knee	4	Electric motors/cable driven	Physical therapy	Active /assistive/resistive	Motion	CP	No	Clinical trial
Paediatric ICARE (2011) [197, 198]	Hip/knee	2	DC motors	Physical therapy	Active/assistive/resistive	Speed	Neurological	Yes	Feasibility study
Rutger ankle CP system (2011) [112, 199]	Ankle	6	Pneumatic	Physical therapy	Active/resistive	–	CP	Yes	Clinical trial
SS-POINT (2013) [135, 200]	Ankle	2	DC motors	Physical therapy	Passive/active/resistive	–	Neurological	No	Clinical trial
TPAD (2014) [102, 103]	Hip/knee	6	AC motors	Physical therapy	Assistive/resistive	Motion	CP	No	Clinical trial
Pedbot(2016) [153, 201]	Ankle	3	DC motors	Physical therapy	Active/assistive/resistive	Position	CP	Yes	Clinical trial
Wyss Institute (2017) [202]	Hip/knee	8	DC motors/cable driven	Physical therapy	Assistive	Gait segmentation/motion/force	CP	Yes	Prototype
Pro-Gait (2017) [40]	Hip/knee	2	DC motors	Physical therapy	Passive		CP	Yes	Prototype
UFMG (2017) [87]	Hip/knee	2	Electric motors	Physical therapy	Passive	–	CP	Yes	Prototype
Leg Press (2017) [89]	Knee	2	Linear electrical motors	Physical therapy	Assistive/resistive	Impedance	Neurological	Yes	Prototype

**Table 6:** Lower limb exoskeleton rehabilitation robots

System (year)	Treated part of the body	DOFs	Actuator	Type of rehabilitation	Type of training	HCI input	Paediatric disease (design for or treated condition)	Paediatric design	Stage of the device
Lokomat (Hokoma, Switzerland) (2007) [203, 204]	Hip/knee	4	DC Motors	Physical therapy	Passive/active/assistive/	Impedance	Neurological	Yes	Clinical trial/commercial (FDA)
HAL (Cyberdyne, Japan) (2007) [91, 129]	Hip/knee/ankle	6	DC Motors	Physical therapy/assistance	Assistive	Footswitch EMG	CP	Yes	Clinical trial/commercial (FDA)
HWA (Honda, Japan) (2007) [205, 206]	Hip	2	DC Motors	Physical therapy/assistance	Assistive	Movement	CP	No	Clinical trial/commercial
University of Verona (2011) [207]	Hip	2	Pneumatic	Assistance	Assistive	Footswitch	CP	Yes	Feasibility study
Ekso-GT (ekso Bionics, USA) (2012) [127, 208]	Hip/knee	4	DC motors	Physical therapy	Passive/assistive	Hip movement	ABI	No	Clinical trial/commercial (FDA)
PediAnklebot (2015) [75, 118]	Ankle	2 active + 1 passive	DC motors	Physical therapy	Active/assistive	Impedance	Neurological	Yes	Clinical trial
Walkbot K (P&S Mechanics, South Korea) (2016) [209, 210]	Hip/knee/ankle	6	AC motors	Physical therapy	Passive/assistive/active	Impedance	Physical disabled	Yes	Clinical trial/commercial (FDA)
Robogait (Bama teknoloji, Turkey) (2017) [211, 212]	Hip/knee	4	Electric motors	Physical therapy	Assistive	Force	Physical disabled	No	Clinical trial/commercial
WAKE-Up (2017) [17, 213]	Knee/ankle	4	SEA	Assistance	Assistive	Footswitch	CP	Yes	Feasibility study
Universidad Pontificia Bolivariana (2017) [214]	Hip/knee	4	DC motors	Physical therapy	Passive	–	Physical disabled	Yes	Prototype
CPWalker (2017) [92, 154]	Hip/knee	4	DC motors	Physical therapy	Passive/active/assistive	Impedance/EEG/LRF	Neurological	Yes	Clinical trial
Rehabilitation Institute of Chicago (2017) [94, 215]	Ankle	1	DC motor	Physical therapy	Passive/assistive/resistive/active	Torque/position	ABI	No	Clinical trial
ATLAS (2017) [146, 167, 216]	Hip/knee/ankle	10	SEA	Assistance	Active/assistive/passive	Footswitch/position/force	SMA, SCI	Yes	Clinical trial
P.REX (2017) [95, 100]	Knee	1	DC motor	Physical therapy/assistance	Assistive	Footswitch/position/torque	CP	Yes	Clinical trial
University of Arizona ankle (2018) [73]	Ankle	1	DC motors/cable driven	Physical therapy	Assistive/resistive	Footswitch/torque	CP	Yes	Clinical trial
Tsukuba University (2018) [74]	Knee	2	Electric brake	Assistance	Assistive	Footswitch	CP	Yes	Feasibility study
Los Olivos University (2018) [217]	Hip/knee	4	DC motors	Assistance	Assistive	Joystick	DMD	Yes	Prototype
P-Legs (2019) [71]	Hip/knee/ankle	6	DC motors	Physical therapy/assistance	Passive/assistive	Impedance	Neurological	Yes	Prototype
ExRoLEG (2019) [218]	Knee	2	Linear actuators	Physical therapy/assistance	Assistive	EMG/limit switch	CP	Yes	Prototype
Cleveland State University (2019) [176, 219]	Hip/knee	4	DC motors	Physical therapy/assistance	Assistive	Position velocities	CP	Yes	Prototype
ExoRoboWalker (2019) [220]	Hip/knee/ankle	6	DC motors	Physical therapy	Passive		CP	Yes	Prototype
Indian Institute of Technology Guwahati (2020) [49]	Hip/knee/ankle	6	DC motors	Physical therapy	Passive		Physical disabled	Yes	Prototype
Instituto Politécnico Nacional (2020) [221]	Hip/knee/ankle	6	DC motors	Physical therapy	Assistive	EMG	Physical disabled	Yes	Prototype

**Table 7:** End-effectors rehabilitation robots for upper and lower limbs

System (year)	Treated part of the body	DOFs	Actuator	Type of rehabilitation	Type of training	HCI input	Paediatric disease (design for or treated)	Paediatric design	Stage of the device
KPT Cycla (Kinetec, France) (2010)(96)	Upper: shoulder/elbow Lower: hip/knee	2	Electrical motor	Physical therapy	Passive/active	–	DMD	No	Clinical trial/commercial (discontinued)

In respect of the developmental stage of the devices, the stages were classified into four categories: (1) commercial in the case the robots are available for its commercialisation; (2) clinical trial when the robot undergo a study where the participants were assigned to groups undergoing similar forms of therapy, but at different intensities, using various devices or undergoing various forms of therapy in a different order, aiming to determine the efficiency of therapy [28]; (3) feasibility study when the experiments conducted with a low number of people, often using the prototype of a device, to evaluate its safety and clinical feasibility without showing the potential benefits of the device [28]; (4) prototypes when the robots had not performed any test that involves people. It can be observed that 18 of them reached the commercialisation phase, but only 9 of them are certified by the US Food and Drug Administration (FDA). However, only 5 of the commercially available devices present a paediatric version of the rehabilitation system. In the case of clinical studies, 34 systems conduct at least one clinical trial, 9 presented a feasibility study, and 15 are in the prototyping phase.

From the 58 devices, it was apparent that the majority (67%) were designed or had been redesigned for children. When it comes to the type of robot, more than half were an exoskeleton type structure. In the past five years, there is a trend (Tables 3, 4, 5, 6, 7) for this structure to be more popular with designers than end-effectors for this structure robotic rehabilitation in paediatrics. Additionally, it can be noted that the majority of robots not explicitly designed for children are end-effector devices. In the case of the exoskeletons, the degrees of freedom (DOFs) are related to the number of joints and limbs that are powered. Therefore, it is possible to find exoskeletons with passive DOFs, which means that those joints are not actuated, but allow the free movement of the children´s joint. In contrast, for the end-effectors, the relation of DOFs of the robot and the actuated joints is not linear and depends on the robot’s mechanical design.

### Actuators

Rehabilitation robots are moved by devices called actuators. Actuators convert a source of energy (e.g. electrical, thermal, pneumatic) into mechanical motion. Commonly rehabilitation assisted robots are powered by electrical actuators. Among the compared systems in Tables 3, 4, 5, 6, 7, over 93% of the robots used electric motors as the actuator, and about 7% used pneumatic actuators.

#### Motor actuator

In robotic-assisted rehabilitation, the most common actuators are electrical motors with a rigid power transmission element such as a harmonic drive, ball-screws, timing belts, and chains. Unfortunately, their need for transmission negatively affects the back-drivability, efficiency, safety, size, and mass [97]. Nevertheless, they were likely chosen since they are efficient and easy to control. Some examples of paediatric robotic rehabilitation devices using electrical motors and rigid transmission are the Pedianklebot that used two brushless dc motors and a Rohlix linear traction device [98], the electric motor with timing belt used in ChARMin [99], or the motor with chain transmission used in P.REX [100].

Some researchers [64] used a cable-driven transmission to replace the rigid transmission for an elastic cable to improve the power to weight ratio and lower the inertia over the treated body segment. Examples include the ankle exoskeleton designed by The University of Arizona [101] or TPAD [102], an end-effector robot for gait rehabilitation that used Bowden cables attached to the hip to generate assistive forces. This change in the transmission brings other advantages like modularity, simple architecture and is convenient for reconfiguration, even though they present some disadvantages being unidirectional and difficult to model and control [97, 103, 104].

Following the concept of adding a flexible element in series with the actuator to improve the electric motors’ compliance, serial elastic actuators (SEAs) incorporate an elastic part in series with the electric actuator. This elastic element helps to decrease the actuator’s impedance and inertia and increases the back-drivability allowing better force control, even though they are limited by a large volume, heavy mass and complicated structure [17, 105]. An example of the use of this technology is the ATLAS exoskeleton [106].

#### Pneumatic actuators

Some authors considered that the mechanical linkage of the electric actuators is too heavy and can generate resistance at the joints, making them inadequate for rehabilitation applications [74, 107]. Instead of electrical motors, they used pneumatic actuators, consisting of a simple air pressurisation mechanism in an expandable chamber, converting the energy from the compressed air to mechanical motion [18, 108, 109]. Their main advantage is improved back-drivability, and they are often lightweight at the site of actuation, have high power density, and can generate fast movements. They are not without limitations; firstly, poor portability because they need external compressors or fluid tanks as the power source. Secondly, it is challenging to create a good model and control strategy due to their nonlinear response to input pressure [109–111]. Among the devices analysed that used pneumatic actuators were the Rutgers ankle platform [112] for CP children and two gloves for hand rehabilitation PneuGlove [113] and Gloreha [33].

### Training strategy

Devices for robotic rehabilitation may provide different training strategies depending on the type and severity of the patient’s impairment. These can be divided into passive, active, assistive, or resistive [42]. In general, the devices can offer more than one type of training.

In passive training, the force/motion is generated by the robot alone to perform the exercise. The advantage of this training is that patients with minimal muscle activity can receive therapy. For instance, through repetition of a movement, ROM can often be maintained with muscles and joint structures (e.g. ligaments) repeatedly stretched, ultimately maintaining their physiological length. Such movement reduces contractures at joints, which can finally be very useful to caregivers making a notable difference to the ease of transfers (e.g. sitting in a wheelchair to lying in bed). Examples of devices using passive training are Innowalk [114] and Intellistretch [115].

In the active training mode, the patient’s muscle can still generate activity on the affected limb. The robot does not help, making the patients perform the exercise by themselves at least partially. The active mode provides data concerning torques and the ROM produced, allowing assessments before and after therapy/surgery. For instance, Kinarm [116] and Lokomat [117] are devices that can perform active training.

For assistive or active-assistive training, the muscles of the affected body part can still be activated. Therefore, the patient can at least partially perform the exercise or movement without the robot. The assistance will be triggered after a particular event is detected through an HCI, allowing the patients to move further with the robot’s help. Assistive training is relevant as it involves the active participation of the children. Moreover, it improves the physiological responses needed to maintain and increase muscle strength and length, ultimately leading to improved ROM, in which the muscles provide some of the torque required. Due to these advantages, many designers have produced devices that use this training mode. Examples are Pedianklebot [118] and the wrist-robot [119].

As the name implies, the robot applies a force opposing the desired movement in resistive training, making the task more challenging. Resistive training is used to enhance muscle strength in the treated limb. This type of training was employed in the ankle device developed by the University of Arizona [120] and the upper limb end-effector NJIT-RAVR [121].

### Human–computer interface (HCI)

The term HCI refers to methodologies to identify the user’s intent to move in the desired direction from different input sources and translate this intention into a command for the robot to move to facilitate the appropriate actions [122]. The designers who report upon the use of an HCI have primarily developed assistive training. Two main types of HCI inputs were identified: those associated with physical interactions and physiological signals [123]. In this aspect, the devices can rely upon only one signal as the input source or use two or more signals as input to start the desired movement.

The main physical interactions used on HCIs to control such robots are Impedance/admittance, body-powered control, and gait phases detectors. Impedance and admittance control are the two most commonly used HCI. They are based on the relation between position and force rather than controlling either force or position explicitly. Impedance control accepts position or velocity as the input and outputs force or torque, and admittance is the opposite of impedance. Hence, force or torque are inputs, and velocity or position the outputs. This method could provide a natural, comfortable, and safe touch interface [122]. Some examples of devices that used this HCI are the NJIT-RAVR [124] and Rehaptic [125] upper limb robots that employed admittance control or the robots for upper and lower limb Inmotion2 [126] and Pedianklebot [118] that applied impedance control.

When the children cannot generate an intention to move with the treated limb, body-powered control is applied. It consists of using the movement of a different body part as the trigger signal to initiate the rehabilitation robot. The main drawback of this approach is that it is hard to control many degrees of freedom due to the activation system’s simplicity. An example of this HCI is the Ekso robot, where the activation was made by moving one’s body weight laterally and then forward to trigger the assistance [127].

In assisted gait, a favoured approach for HCI is the use of gait phase detection. This technique identifies the different gait phases (heel strike, midstance, toe-off, and the swing phase) to apply forces to assist the children’s movement depending on the gait phase. Robots usually perform gait segmentation using inertial measurement units (IMUs) to detect angular velocities of the shank and/or the thigh, or footswitches to detect the foot’s ground reaction forces while the child is walking [128]. The main advantage is that splitting the gait cycle into discrete phases provides enhanced consistency and robustness to an inherently variable process and allows lower-level controllers’ implementation within each phase. The problem is that gait detection should be characterised for every target group, as the physical disability modifies the gait pattern [100]. An example of this type of system is the P.REX exoskeleton which utilised a combination of the footswitch and IMUs to detect the different gait phases to provide different levels of assistance within each phase [100].

Alternatively, for HCI based on physiological signals, Electromyograms (EMG) that measure electrical activity in the muscles and electroencephalograms (EEG), which measure electrical activity in the brain, are the main signals used. They are widely utilised because they can be obtained using non-invasive techniques without the need for medical intervention.

Concerning electromyograms, the primary type is surface electromyography (sEMG), a non-invasive and easy-to-configure procedure in which adhesive electrodes are placed on the skin above the muscle of interest. The benefit of using the EMG signal is that it allows detection of the user’s intent before the movement occurs. The electrical activity can be detected even if it is insufficient to generate movement of a joint. However, sEMG can suffer from contamination of the signal by electromagnetic interference, skin perspiration, movement of electrodes and crosstalk artefacts. Also, for each muscle group of interest, a single EMG channel only shows the activation of that group. So, to perform an activity where many muscles fibres are recruited, it is necessary to use multichannel sEMG. Some examples of this technology in paediatric rehabilitation robots are the lower limb exoskeleton HAL [129] and the device for upper elbow rehabilitation of the San Juan National University [130].

The electroencephalogram (EEG) signal is recorded using many small surface electrodes, often configured in a bathing like cap placed over the scalp that detects the underlying electrical signals. The main advantage of the EEG signal is that the physical disability level does not limit it. Even if the patient has lost all their ability to move the limb required for a task, the brain activity thought to be related to the intent to activate the muscles can be recorded. There are two main disadvantages to this system. Firstly, it is unsuitable for children with brain damage as they cannot generate standard brain patterns for limb activation. Secondly, the EEG signal has greater variability within it than the EMG signal, and it is also easily affected by changes in the patient’s mood and attention. Examples of the EEG signal use are the CP walker that used this signal as a part of its HCI to help children with a physical disability move their legs [120] and the Exohand-2 that used the EEG signal to interact with the exoskeleton [131].

### Treated conditions

The majority of studies and devices were for children with neurological conditions (np = 183, 89%), CP being the most studied condition (np = 129, 63%). In contrast, other neurological disorders included ABI and strokes. Significantly few researchers investigated other conditions such as neuromuscular diseases (np = 15, 8%) and traumatic injuries to limbs and the spine (np = 6, 3%). The results obtained from the studies that perform clinical trials or a feasibility study suggests that robotic rehabilitation could benefit children with physical disabilities.

#### Neurological disorders

Concerning CP, there was evidence of improvement in physical disability using assisted rehabilitation robots. The benefits include an increase in muscle activity [120, 132], endurance for physical activities [133, 134], improvements of balance [114], walking speed [134, 135], the strength of the muscles [136, 137], ROM of the joints [84, 138], upper limb kinematics [139], and manual dexterity [33, 140].

For paediatric ABI, there were reports in the improvement of the walking ability [141], improvement of the lower limb motor performance [94], increase in the ROM of the wrist joint and force increase in the hand [142], improvement in motor function, and gait pattern [143, 144].

In children who suffer a stroke, three studies used rehabilitation robots while performing physical therapy. Marini et al. [119] demonstrated an improvement in wrist motion after the robotic therapy, and Bützer et al. [26] showed the possibility of using a wearable hand exoskeleton to assist children during task-oriented training could be helpful for rehabilitation therapies or assist children during ADLs.

#### Neuromuscular diseases

The neuromuscular disease presented a different scenario than neurological disorder due to the degeneration of their muscles as the disease progresses, making hard the use of rehabilitation robot due to stiffness in the robot’s joints, which can harm children’s weak muscles. Hence they require compliant actuation [145, 146]. Jansen et al. [145] found that robotic rehabilitation therapy on upper and lower limbs help prevent functional deterioration in children with DMD. Meanwhile, Ganguly et al. [147], Garcia et al. [148] and Sanz-Merodio et al. [146] showed an improvement in walking ability in children with SMA with the assistance of ATLAS, and the exoskeleton was designed to provide Robotic-assisted gait training for children with SMA. Moreover, Koo et al. [149] reported improved arm mobility in children with DMD while using a robotic arm device.

#### Traumatic injuries

Even if traumatic injuries are common in the paediatric population, robot rehabilitation has not been applied widely in injuries that differ from those at the head. Only scarce information was found related to these conditions. A study of hands robotic rehabilitation was found, highlighting the possibility of using robotic devices to treat burns [150]. Additionally, a study observed a significant improvement in the arm movement and elbowed angle after physical therapy using an upper-limb exoskeleton for 3 months in children that suffer a car accident [130]. Finally, another case reported improving walking ability after robotic-assisted gait training in a girl with SCI [151].

## Discussion

It is possible to see that various novel rehabilitation robots have become available to rehabilitation professionals and clients in recent years. And this trend will continue as is possible to incorporate them in activity programs aimed at improving independent function [34–37] where they offer advantages over the traditional rehabilitation therapies, as they reduced the required effort of therapists during the exercises of the therapies, allowed massed practice in children with substantial limitations and provide information of the patient. Furthermore, they have the potential to be used as assistive devices to aid functional performance for users when they are worn. These possibilities will lead to a new variety of ways for assessment and intervention impacting users’ abilities, task demands, or the environment to promote functional performance and participation.

The findings of this review indicate that the design and development of robotic technologies for the physical rehabilitation of children is in a preliminary stage of development, as many of the devices were designed for adult patients. However, there is a trend toward creating robots specifically for children [17, 26, 57, 148]. Yet less has been done to prove the benefits and constraints of such a system.

Traditionally, rehabilitation robot designers have focused solely on improving physical function [58], which can lead to rejection of the devices as not all the needs of children with disabilities are considered. Thus, to ensure successful adoption of the technology, the rehabilitation robots should cover these needs of the children. Hence, stakeholders’ cooperation is essential through their integration within the design and production process by providing feedback. Designers can use this feedback to validate that the robot meets the stakeholder’s needs. However, the fulfilment of these needs has strong relationships to the chosen technology, mainly the type of robot, the actuator, the training strategy and the HCI. Thus, it is essential to know the advantages and disadvantages of the technology.

### Type of robot

When it comes to the type of robot, we can see a trend to migrate from end-effector to exoskeletons. However, most of the devices that had performed clinical trials were end-effector robots designed for adults. This relation could be because the end-effector robot works on the distal part of the limb, guiding the children limb through a movement [152]. This property is helpful in the case of operability as it does not require adaptation to match the children limb’s size, making it easy to be used by a diverse group of children. Furthermore, the bulky frames over the patient limbs are avoided, helping to reduce the weight that the children need to handle. These advantages come with the problem of the systems requiring bulky and heavy external structures, reducing the device’s portability, constraining its use to medical facilities or specific spaces inside a building. Thus, limiting the amount of therapy that the children can have [153]. Additionally, the activity is restricted to a workspace constraining the number of possible movements [40], which could reduce motivation.

On the other hand, exoskeletons work in parallel to the patient limb to perform the activity. Hence, they can be portable devices with the possibility to provide assistive help during activities of ADLs and robotic-assisted rehabilitation therapy in a single device [26]. This advantage will help to provide free movement to enhance the subject’s motivation and autonomously practise their movement training for longer periods [17, 154]. Furthermore, as technology advance, this freedom in mobility will help to increase the participation of children with physical disabilities in different social activities [155]. However, as the technology moves from clinical facilities to open spaces and robots interact more closely with the children, designers will face notable challenges (e.g. the irregularities of the surfaces on which one walks and how the robot reacts to perturbations outdoors environment). Consequently, the requirements of weight, comfort, safety, portability and social acceptability for the exoskeletons will be harder to achieve.

### Actuators

The paediatric robotic rehabilitation technology is moving from end-effectors to exoskeletons due to their versatility to be used as a rehabilitation tool or an assistive device [26]. Consequently, actuation technology starts to be a critical part of the design as it negatively influences the weight and the size of the robot.

For the end-effectors robots, actuators are not as critical as with the exoskeletons because they could be placed in external structures. This advantage makes it possible to use bulky and heavy actuators like electric motors. However, using electric motors is hard to achieve compliance that is an important property to increase safety as it is needed to avoid opposing forces that can injure the children. In end-effector robots, compliance was achieved using sensors and a control strategy [146] or using a soft material like the Bowden cables [103].

On the other hand, for exoskeletons, the robot design requirements are hard to enhance with the currently used technology. The actuation system components such as motor and rigid elements are designed for industrial applications not to interact with and to be worn by children. However, they are still the standard as they have the advantages of efficiency, are easy to control, and are readably available in the market. Therefore, the choice of the actuation system is crucial to improve the weight, portability and safety of the exoskeleton.

The first exoskeletons relied on electric motors with rigid transmissions, making them bulky and heavy, reducing their compliance as they generate high resistive torque from the metallic links of the exoskeleton. Therefore, making it difficult to move and less safe can cause non-desirable inertial movements [156, 157]. Furthermore, they require external structures to manage the weight of the exoskeleton.

As the rehabilitation robots move from rehabilitation therapies inside a medical facility to assist the children during ADLs, new actuation technology is needed. This challenge led to using SEA actuators and cable transmission since they have the advantage of been intrinsically complaints as they incorporate soft materials, making the device safer.

Using Bowden cables in the transmissions brings other advantages like simple architecture, low weight on the limb’s distal part, and easy to reconfigure. This last advantage is significant in paediatric rehabilitation as it allows to change the motor easily depending on the abilities and size of the children [26, 73], even though they present some disadvantages because they become unidirectional and difficult to model and control. Instead, in the case of SEAs, which still require rigid links, they were highlighted on the use for children with neuromuscular diseases, as some children are not only weak on the affected joint but the entire body. Thus the exoskeleton must hold the children, but at the same time being compliant to avoid inertial forces that can harm the weak muscles of the children [146].

Another type of actuator used on the robots was the pneumatic. Their attributes of low weight and easy-to-manufacture actuators of different shapes and sizes [158, 159] make them a desirable technology in this field. They are easy to adapt to children with various conditions. However, their main constraint is that they are typically connected to external mechanisms like compressors and pumps cumbersome and noisy. Thus, reducing their portability and appealing making them impractical to use outside clinical facilities.

In Table 8, the advantages and disadvantages of the current actuator technology are presented. It is possible to notice that there is no perfect actuator technology, so more research in this area is needed. Moreover, in the future will be interesting to see devices that use different soft actuators technologies that are inherent compliant and lightweight, such as the already mention SEAs, pneumatic, and Bowden cables. But also new technologies that are under research to be used on rehabilitation robots, like shape memory alloys [160], dielectric elastomer [161], or twisted and coiled polymers actuators [162], as they will reduce the overall weight and increase the compliance. Furthermore, this new technology can be manufactured in different sizes and shapes [163] that could be easily adapted to robots for children of varying height and ability conditions.

**Table 8:** General summary of advantages and drawbacks of each actuation technology

Actuation technology	Advantages	Drawbacks
Electric motors	High precision Easy to control Readably available in the market	Not compliant Large size Heavy Noisy
SEA	High precision Easy to control Compliant Better force control	Large size Heavy Complex structure
Bowden cable	Modularity Simple architecture Easy to reconfigure Low weight on the distal part of the limb	Unidirectional Difficult to model and control
Pneumatic	Lightweight Compliant Have high power density Fast actuation Low cost Easy to manufacture in different shapes and sizes	Poor portability because they require external components Difficult to model and control

### Training strategy

In the case of the training strategy, there is no best strategy, but it rather depends on many factors like the abilities and disease that the children have. For example, passive training is suitable for patients with limited mobility; however, when the children are able to generate movements, it tends to decrease children’s participation during the exercise, thus reducing the efficiency of the training [164]. That is why most of the research on the training strategy is centred on assistive training, where the children’s participation is needed. This engagement with the therapy increases the motivation of the children to perform the activities, enhancing the benefits from the therapy [165]. Another advantage of assistive training is that it is used together with video games to increase children’s motivation and social interaction [166]. In addition, this strategy is required for assistive devices. It needs to provide the required intensity to generate the movement safely, efficiently, and reliably, depending on the applied force by the user [167]. However, there is no clear which is the best strategy to provide assistive movement, where some examples of different assistive strategies are guidance force, path control, and locomotion strategy [57, 154, 165, 166].

Alternatively, some researchers suggest that resistive training could be more beneficial for rehabilitation therapy than an assistive force, as it increases the engagement of the children, which can help drive motor learning [120, 168]. Hence, further research is required on the optimal training strategy to increase the benefits from the rehabilitation therapies.

### Human–computer interface

The HCIs are essential in developing robotic rehabilitation robots, as they are the medium for the interaction between the children and the robot, impacting the functioning of the actuators and training strategies directly. Thus, HICs are a crucial factor for safety and motivation needs, as it is how the children “communicate” their intention to the robot. Consequently, If the HCI is complex to use, it could lead to the rejection of the device [52]. Furthermore, for safety reasons, the HCI must detect the trigger signal properly and discern between intended movements and involuntary movements, as it can generate undesirable responses [41, 169]. For instance, in the case of CP patients presenting increased muscle tone, rapidly occurring muscle spasms, and severe jerks, which can be considered as a deviation of pattern, causing the robot to apply undesired forces to correct for it or turn off the device suddenly [170].

Consequently, selecting the best HCI for every case needs to be evaluated depending on the capabilities of the children. For example, it could be challenging for patients with advanced muscular dystrophy to use EMG and admittance/impedance interfaces as their muscles progressively deteriorate, turning unable to activate the muscles to generate a movement or a detectable signal [149, 171]. On the other hand, EEG could lead to a better motor function recovery for children with CP as it integrates the central nervous system into rehabilitation therapy [154]. However, this technology could be hard to implement in patients with a cognitive deficit, requiring concentration [131, 172]. For HCIs, it would be interesting to see more devices using physiological signals as they can also evaluate the efficiency of the therapy [154], novel approaches of body-powered control to address children with limited mobility of limbs [173], and devices that integrate different HCI strategies to make the system more robust and adaptable [71, 167, 174].

### Treated condition

To better understand how the technology can positively impact children’s lives, it is essential to analyse how the rehabilitation robot’s technology has addressed the different paediatric conditions that can generate physical disability. Because, even if they share in common the deterioration of the musculoskeletal system, each one of the conditions presents certain specific characteristics that need to be considered.

Most of the research has focused on children with neurological conditions, particularly children with CP. However, it seems likely that many of the designed robots that currently work with neurological conditions could also be utilised in traumatic injury scenarios, especially because they have been designed for rehabilitation therapies that can improve common problems across both neurological and traumatic injuries like ROM deficits and a lack of ability to generate muscle force [80, 150]. Contrarily, neuromuscular diseases present a different scenario than neurological disorders and traumatic injuries as the diseases are progressive, making it hard to obtain a permanent improvement on the children skills. Thus, the designs have been focused on design devices capable of assisting with exercise and helping with the ADLs to maintain specific abilities (e.g. walking) for a longer period of time [122, 155].

Unfortunately, the outcomes proving the efficiency of rehabilitation robot in children is still scarce, as the pieces of evidence are low and weak. Hence, the information coming from these studies should be asses carefully, as there are very few randomised controlled trials, with small sample sizes and variability in children’s ability, outcomes measures, treatment protocols, and used devices [31, 65]. Thus, to better understand if the designed robots fulfil the paediatric needs properly by improving their quality of life and physical ability, more studies and robots designed especially for them are needed. Furthermore, more studies with children presenting different conditions from neurological ones are needed, as it can be noticed that the treated condition impacts the requirements design of the rehabilitation robot.

### Limitations

It was apparent that some studies were conducted with participants from a wide range of ages; therefore, it was difficult to target all the articles that include paediatric participants. Another problem was the upper bound on the paediatric population’s age as some papers with the term young adults included paediatric participants.

## Conclusion

While robotic rehabilitation is gaining momentum with increasing numbers of devices being produced for adults, there is a lack of well-designed and effective products available for children. Early examples of robots have often been created by scaling downsize to meet the smaller stature of children. Few robots have been specifically designed and produced, with children being the focus of the project/program. It is apparent that children have special needs, and these need to be incorporated into designs early in the development program. And even if the fulfilment of these needs is closely related to the chosen structural and technological components like the actuator, training strategy and HCI, they go beyond them. Consideration must also be given to the aesthetics that appeal to children and the need for the robot’s structure to be as unobtrusive as possible. Without such needs being met, no matter how effective the robot works from an engineering perspective, it will not be utilised well by the child.

It is apparent that there is still a lack of understanding of what the most effective therapy is to improve function and quality of life in specific paediatric conditions (e.g. CP or Stroke). Nevertheless, common impairments (e.g. ROM, strength) must be addressed across numerous clinical conditions if patients improve function in everyday tasks. Hence, there is much opportunity for robots to play a role in assisting paediatric rehabilitation. A much more difficult goal to achieve is the development of robots to assist children. At the same time, they do function-related tasks like walking, sitting, lying, and assisting when the child moves from one posture to another. This demand increased complexity throughout the various engineering systems of the robot. After that, a further challenge lies in the robot being able to assist indoors within a relatively safe environment and outdoors where the “lay of the land” is notably different and less predictable. Hence, exploring new technologies to actuate the system and detect children’s intentions when they want to move is necessary.

## Data Availability

Not applicable.

## Abbreviations

WHO: World Health Organization
FDA: US Food and Drug Administration
CP: Cerebral palsy
ABI: Acquired brain injury
DMD: Duchenne muscular dystrophy
SMA: Spinal muscular atrophy
SCI: Spinal cord injury
np: Number of papers
ROM: Range of motion
ADL: Activities of daily life
HCI: Human–computer interface
EMG: Electromyography
EEG: Electroencephalogram
LRF: Laser range finder
DOF: Degree of freedom
AC: Alternate current
DC: Direct current
SEA: Serial elastic actuator
